# Assessment of Awareness and Knowledge on Novel Coronavirus (COVID-19) Pandemic among Seafarers

**DOI:** 10.3390/healthcare9020120

**Published:** 2021-01-25

**Authors:** Gopi Battineni, Getu Gamo Sagaro, Nalini Chintalapudi, Marzio Di Canio, Francesco Amenta

**Affiliations:** 1 Telemedicine and Telepharmacy Centre, School of Medicinal and Health Products Sciences, University of Camerino, 62032 Camerino, Italy; getugamo.sagaro@unicam.it (G.G.S.); nalini.chintalapudi@unicam.it (N.C.); francesco.amenta@unicam.it (F.A.); 2 Research Department, Centro Internationale Radio Medico (C.I.R.M), 00144 Rome, Italy; mdicanio@cirmservizi.it

**Keywords:** COVID-19 spreading, online survey, awareness and knowledge, ships, seafarers

## Abstract

*Background*: The ongoing pandemic due to the novel coronavirus (COVID-19) is becoming a serious global threat. Experts suggest that the infection can be controlled by immediate prevention measures. Sailing is one of the occupational categories more vulnerable to this virus outbreak due to the proximity of the working conditions. *Objective:* Awareness and knowledge assessments of seafarers towards the current epidemic is mandatory to understand the effectiveness and success of the infection control measures adopted by shipping companies. *Methods:* In this study, we presented an online questionnaire survey to determine the knowledge levels of COVID-19 among seafarers. The data were collected by self-reported survey, and analysis was done by the analysis of variance (ANOVA). The *t*-test was used to understand the knowledge attitude differences to COVID-19 among different occupational groups of seafarers, and the *p*-value ≤ of 0.05 was considered statistically significant. *Results:* Among 1,458 responses received, 92.82% had a college or university degree. The results reported that the mean COVID-19 knowledge score was 5.82 (standard deviation = 0.51, range 0–6), and the overall correct percentage was 97%. There was a statistically significant difference between age groups (F (4, 1453) = 5.44, *p* < 0.001) and educational groups (F (4, 1453) = 1.52, *p* < 0.001). The knowledge score was not significantly different across the educational status of the participants (F (2, 1455) = 1.52, *p* = 0.220). *Conclusions*: The present study highlighted good knowledge and behaviours among sailors about COVID-19. However, shipping companies need to come up with new campaigns to hold optimistic practices and suitable guidelines on ships, including cruise boats, to keep sea workers always alert and collaborative in mitigating the spread of COVID-19.

## 1. Introduction

The novel coronavirus disease, or COVID-19, was first identified in December 2019 at a Wuhan wet market in China and then constantly spread all over the world at a rapid pace [1]. As of 5 January 2021, more than 85 million cases have been reported, including 1.86 million deaths [2]. In many cases, COVID-19 develops mild-to-moderate symptoms. In some cases, it might cause severe sickness, including pneumonia and, consequently, death. A person who is infected by the virus usually takes five to seven days to develop symptoms, and it can extend up to 14 days [3].

Currently, many European countries like Italy, France, Germany, and others have been exposed to the second wave of the COVID-19 pandemic [4]. These countries were severely hit by the first wave of pandemic during the spring, which was followed by the second wave during late summer and autumn. Epidemic data present the virus characteristics, and its effects are varied between these two periods. The symptoms like pneumonia, dyspnea, fever, cough, chronic neurological diseases, and type 2 diabetes mellitus are often found in both waves. In severe cases, the symptoms usually get worse gradually after the initial appearance. To slow down the spreading of the virus and reduce its effects, governments around the world have made travel restrictions and closed their country’s borders [5]. Various ports and air terminals are closed, ships’ entries are denied, and all planes grounded.

Due to the limited medical resources, natural exposure to new environments and crowded, enclosed areas make the high risk of the present novel pandemic spread among many cruise ships [6]. On 4 February 2020, the UK-registered ship named the Diamond Princess was exposed to a large outbreak of COVID-19, and this was quarantined for about one month at Yokohama, Japan. More than 700 individuals were infected, including 14 deaths [7]. Over 40 cruise ships have confirmed positive cases of COVID-19 infection onboard, and port authorities and governments are advising people to avoid travelling on cruise ships and restraining ships from docking [8]. Besides, many maritime transport lines have been suspended to prevent the epidemic spread [9]. 

Seafarers are the unsung heroes of this pandemic, because over 90% of the world trade, including medical goods, raw materials, essential foods, and manufactured goods, depends on them [10]. Based on a report published by the International Maritime Organization (IMO), seafarers are collateral victims of this pandemic emergency, as travel limitations have left a huge number of them abandoned on boats or unfit to join ships [11]. Moreover, commercial fishing is a main source of the world’s food. Several sailors are ready for stretched out work timeframes; to maintain a strategic distance from becoming infected, crewmembers need to change all the time, and this includes nearly 100,000 sailors every month [12,13]. 

The COVID-19 pandemic has introduced phenomenal circumstances around the world. The worldwide health authorities have been focused on controlling the disease by mitigating actions to limit the fast-spreading. Currently, two vaccines—namely, Pfizer-BioNtech and Moderna’s—are authorized and recommended to prevent COVID-19; additionally, the presence of more than 50 COVID-19 vaccines are in trials, yet the world is looking for safe and effective ones [14]. Of the problems caused by the COVID-19 pandemic, around 90,000 sailors are now stuck on cruise ships without passengers [15]. Since the ship is a closed environment, there is a high chance of being infected. After being at sea for at least 14 days, and if no crewmember shows the symptoms of the COVID-19 illness, then a ship can be considered as virus-free and, eventually, safe.

The recent literature on COVID-19 highlights scientific knowledge or epidemic projections, especially in the public health environment [16]. Outcomes integrated with scientific knowledge tend to identify the key safety-related issues. Apart from the clinical and healthcare aspects like the safety of medical doctors, social and occupational safety, and, in particular, mental health, they have gained large attention from the COVID-19 scientific community [17]. The individuals living in closed environments like ships should have basic knowledge when addressing key social issues, including an urgent need to understand and believe the science about the COVID-19 pandemic that is shattering the present world. 

This study presents an online cross-sectional survey designed to understand seafarers’ behaviour and knowledge during COVID-19. A similar study was conducted on USA residents during the early epidemic phase to evaluate their knowledge levels and behavioural characteristics [18]. This study is among one of the first attempts to measure the knowledge on COVID-19 among crew members of merchant ships addressing requests for telemedical advice to the Centro Internazionale Radio Medico (C.I.R.M., International Radio Medical Centre). 

## 2. Methods 

### 2.1. Participants

The current cross-sectional investigation enlisted a sample of seafarers from the C.I.R.M. [19]. With more than 100,000 seafarers assisted onboard ships, the C.I.R.M. is the maritime telemedical centre with the largest experience of medical assistance to sailing seafarers. It was established in 1935 to give free radio medical advice to ships of all nationalities navigating in international waters. The C.I.R.M. is the Italian Telemedical Maritime Assistance Service (TMAS). The online questionnaire was delivered to 5000 seafarers, and 1458 (about 30%) agreed to provide consent to participate in this survey. We assumed that the participants that did not show interest in this study was due to a lack of internet onboard, staying with family because of fear caused by COVID-19, or a low-level knowledge of English. 

### 2.2. Survey

The questionnaire included in the survey was refined by phrase changing, the possibility of adding new questions, and modifications of other research studies that provide extensive knowledge on the COVID-19 epidemic. Two C.I.R.M. doctors thoroughly reviewed the final questionnaire to make sure each item of the survey was clearly understood. The final questionnaire consisted of 25 questions and was distributed through a Google Form link. 

The survey was organized into three sections: demographic characteristics, including age, rank onboard, members onboard, and educational status, etc. (5 items); personal characteristics (14 items); and the knowledge questionnaire had 6 questions regarding clinical presentation (2 questions), COVID-19 transmission routes (2 questions), and prevention and control (2 questions). Moreover, the questionnaire was adapted from previous studies on COVID-19 knowledge [18,20]. These questions were answered by correct and incorrect options. As a result, a correct answer was assigned “1”, and an incorrect answer was assigned “0”. 

Before starting the survey, seafarers read an informed consent explanation that portrayed that cooperation was deliberate and that they could stop whenever. By tapping on a “next” button, members were considered as agreeing to finish the online questionnaire. The survey consisted of closed-ended questions, of which six permitted the seafarers to have the chance to give further details if the “other” alternative was chosen from the multiple-choice questions. The closed-ended questionnaire consisted of categorical, dichotomous, multiple-choice, and Likert-type questions on five-point rating scales. At the end of the questionnaire, we requested the participants provide final feedback regarding their participation in the study. 

### 2.3. Reliability and Validity of Responses

The main objective of any questionnaire is to gather relevant information most reliably and validly. These factors are commonly associated with the conduction and selection of valid research instruments. As mentioned, this was a study related to onboard behavioural characteristics of seafarers, and we adopted the face validity method that was done by an analysis of the data using Cohen’s Kappa Index (CKI). A kappa value that was greater than 0.6 was accepted as a valid question [21]. The items in the knowledge questionnaire were further validated with the CKI scale. 

### 2.4. Statistical Analysis

Demographic variables such as age, rank, and educational status were done using frequency analysis. Frequencies of correct answers to knowledge questions were determined. A one-way analysis of variance (ANOVA) was used to determine the differences in the mean knowledge scores between the age groups, rank groups, and educational status. The *t*-test was used to understand the knowledge attitude differences to COVID-19 among different occupational groups of seafarers, and a *p*-value of ≤0.05 was considered statistically significant. Statistical analysis was carried out by IBM SPSS v.26 (Armonk, New York, NY, USA). 

### 2.5. Ethical Approval 

The review board members and the Ethics Committee of C.I.R.M. approved this study. The checklist for research ethics during the COVID pandemic was adopted from the UK research integrity office (UKRIO) guidelines [22]. This study was reviewed and approved by the C.I.R.M. Research Ethics Committee (ESI/2020/017). All participants provided consent by responding to a yes/no inquiry toward the beginning of the survey before they responded to the first question. 

## 3. Results

Table 1 includes the participants’ basic demographic characteristics: age group, rank onboard, and educational status. Table 2 presents nine questionnaires that were administered to measure the awareness about COVID-19, including clinical characteristics, transmission, prevention, and control. Among the total number of the respondents, the majority (97.87%) of them reported that they are aware of the novel coronavirus outbreak. Of the respondents that reported, 93.63% said that they were never infected by the new coronavirus, and 88.73% of the respondents reported that none of the people in their immediate social environments were infected. 

Regarding behaviours, 42.57% of the participants reported that they were moving with other staff onboard, and just 0.22% of participants used a bed previously used by someone who got infected. Most seafarers (98.4%) provided a correct response on the transmission of the novel coronavirus. Regarding mental health status, 33 (2.24%) seafarers reported feeling lonely, 862 (59.13%) seafarers reported feeling well, 395 (27.10%) seafarers reported missing family/friends and 168 (11.53%) seafarers reported feeling overstressed. 

The correct answers to knowledge questions ranged from 91.8% to 99.4% (Table 3). The mean COVID-19 knowledge score was 5.82 (standard deviation = 0.51, range 0–6), and the overall correct percentage was 97%. Most of the seafarers (99.4%) were aware of the COVID-19 clinical symptoms, and 95.7% realized that all infected individuals did not develop into severe cases. Viral infections are highly contagious among the people who live nearby and spread by respiratory droplets. Most respondents (97.3%) were aware that COVID-19 can be caused by human transmission when infected persons cough or sneeze. 

Based on the guidelines provided by the World Health Organization (WHO), it is evident that wearing facemasks only can help prevent becoming infected with the virus [23]. In this study, 92% of participants agreed that spreading of the virus can be controlled by wearing masks onboard; 99% mentioned that treatment and isolation are promising ways to reduce the virus transmission, and 99.3% provided the correct response on the incubation period of COVID-19. These findings appreciate the well-known knowledge of emphasizing maintaining onboard social distancing to control further infections. There was a statistically significant difference between the age groups (F (4, 1453) = 5.44, *p* < 0.001) and rank groups (F (4, 1453) = 32.18, *p* < 0.001), as determined by one-way ANOVA. The knowledge scores were not significantly different across the educational statuses of the participants (F (2, 1455) = 1.52, *p* = 0.220) (Table 4).

**Figure 1:**
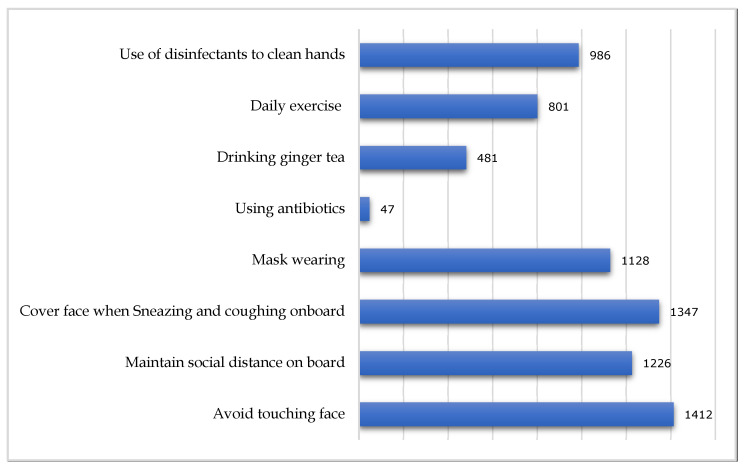
Onboard individual prevention measures by seafarers.

The data on the knowledge of daily preventive measures by seafarers are summarized in Figure 1. As shown, few respondents had limited knowledge due to a low educational status. Among the total respondents, 1412 (97%) indicated that they avoid face touching, 1347 (92.38%) anticipated covering their faces when they sneezed or coughed, and 986 (68%) chose disinfectants for cleaning their hands when soap was not available. Moreover, 1226 (84%) followed social distancing onboard, and 1128 (77.3%) wore masks while moving onboard. To increase their immune systems, 801 (55%) preferred to do exercise, 481 (33%) habitually drank ginger tea, and only 47 (3%) were interested in using antibiotics. 

## 4. Discussion 

Many studies were found related to the knowledge, attitudes, and practices (KAP) concerning the COVID-19 outbreak [24,25,26,27], but the literature search did not identify any works on seafarer COVID-19 knowledge assessments. Due to this, we developed a tool to investigate COVID-19 knowledge, including behavioural characteristics and the necessity of onboard health measures. 

### 4.1. Personal Awareness 

The personal awareness questionnaire was created to understand the factors that decide virus transmission onboard and further classified into three constructs, such as environment hygiene, socio-travel characteristics, and poor health literacy. These, indeed, are considered the four factors that demonstrate seafarers’ knowledge of COVID-19 transmission.

Early data on public health practices embraced to forestall the spread of COVID-19 might help control the virus transmission; it is also necessary to publicize knowledge of the psychological characteristics of the stigma and social discrimination (SAD) in pandemic realities [28,29]. Recent evidence confirmed that the COVID-19 disease is transmitted by either physical contact or respiratory droplets of an infected person [30]. Moreover, contact transmission can be possible when an infected individual onboard touch their nose, eye, or mouth mucosa, and the virus can also be transferred from one surface to another by contaminated hands. Due to this, hand hygiene is mandatory to prevent the COVID-19 virus spread. In this study, 97.3% of seafarers agreed with the concept that the virus spread was caused by respiratory droplets of contaminated individuals. Rubbing hands with alcohol-based soap for at least 20 s is an effective approach to neutralize viruses like corona because of an oily surface membrane that is decomposed by soap [31]. The highest number (93.68%) of seafarers mentioned that they do 20-s hand washes with alcoholic soaps. 

Travel behaviour is another important characteristic of the spread of COVID in any working culture. Due to global trading during the pandemic, the percentage of the population engaged in international travel is higher in Western countries like Europe, the USA, and others. In the present study, 992 (68.01%) seafarers mentioned that they travelled to infected nations during April and May 2020. Moreover, COVID-19 can largely spread among the populations of infected cases in the next social environment [32]. Respondents mentioned that about 58 (4%) people were suspected of contracting COVID-19 during working conditions, and 42 members were confirmed as infected. Maintaining social distancing with other employees also prevents the virus spread, but sometimes, it is hard to avoid staff movements in closed environments. 40% of participants mentioned that they are aware of social distancing but are unable to escape from unexpected situations, and 42.57% are closely moving with others. Maintain isolation onboard for people who are confirmed or suspected by COVID-19. A person in isolation is not supposed to leave the place and keep away from the public on the ship. Others should also be aware of not sharing the belongings of infected people. Most of the seafarers (98.5%) did not intend to use the bed of a COVID-19-infected person. 

Poor health literacy about COVID-19 is an underrated global public health issue. Significant health literacy already seems like an important tool for the prevention of noncommunicable diseases like an ongoing epidemic [33]. In the present research, 97.87% of members said that they were aware of the present virus outbreak, and 98.44% of respondents were well informed about it being caused by human transmission. 

### 4.2. Seafarers’ COVID-19 Knowledge 

COVID-19 is an ongoing pandemic with serious threats to public health [34]. Since most of the vaccines are under trails, preventive measures are the only solution to control it, and therefore, everyone should have minimum knowledge on this novel virus. Attempts to change behaviours are basic in limiting the easy transmission of diseases like COVID-19, and it is unclear whether people knew about the risk of disease and adjusted their behaviours during the early times of the pandemic [35]. Due to its highly contagious nature in enclosed areas, seafarers’ behaviours on a ship are probably the main factor in deciding the spread of a COVID-19 epidemic. Their behaviours are affected by their perceptions and individual knowledge. 

The study outcomes indicated that most of the seafarers were knowledgeable, and respondents achieved a mean of 97% in the knowledge questionnaire. This value is higher than other audiences, like the general public and health workers, which ranged from 62% to 81.4% [24,25,26,27]. In this study, the 92.82% rate of correct answers is probably related to the cultural background of the respondents that mostly had a college or university education. This is also due to the time that the questionnaire distribution happened during the virus outbreak. During this time, seafarers already gained some knowledge regarding COVID-19 prevention and transmission causes via the internet, media platforms, or colleague discussions. The close associations found among educational background, age, and knowledge (*p* < 0.001) supports our claims. On the other hand, the knowledge of daily preventive measures by seafarers was much appreciable. Very few respondents had limited knowledge due to a low educational status. 

In terms of the seafarers’ behavioural characteristics on ships, the respondents presented a positive and encouraging approach towards COVID-19. About 96% agreed that the virus does not develop into a serious illness unless in the presence of other chronic diseases. These inspirational mentalities and high trust in the control of COVID-19 can be clarified by the marine industries’ phenomenal activities and brief reaction times for taking tough control and prudent steps against COVID-19 to defend ship workers and guarantee their better health. These measures include isolation, avoid travelling on other ships, regulations on mask-wearing, and sanitization of the workplace regularly. 

### 4.3. Study Strengths and Limitations 

This is probably the first study that investigated the awareness and knowledge regarding COVID-19 among the seafarer population. The data collection involved nearly 30 shipping companies, including more than 1,000 ship workers. The preliminary results encourage ship authorities to provide explicit guidelines and plan preventive actions to avoid the future spread of the virus at ship working places. Despite the promising knowledge outcomes, the present study had some limitations. Since the data analysis was conducted with the help of a self-reported questionnaire, there is a chance of biased outcomes. Forthcoming works must use administrative questionnaire data to overcome this issue. Besides, community-based sample (like seafarers) studies cannot provide as much evidence on the severity of pandemics as the data collected through participants of author networks. On the other hand, the highest number of participants were from European shipping companies, and future research needs to include worldwide merchant ship members. 

## 5. Conclusions 

The current global pandemic caused by the COVID-19 disease is creating awful situations for both seafarers and marine industries. By maintaining a close relationship between shipping companies, flag and port states, and others, maritime service providers can protect seafarers’ health and, simultaneously, the public [36]. Certain web-based interventions like online questionnaires can enhance the individual knowledge of seafarers by informing, educating, reminding, and monitoring to fight against the ongoing epidemic. Hygiene conditions, waste management, and room sanitation onboard are mandatory to protect an individual’s health during virus outbreaks, including COVID-19. Guaranteeing continuous handwashing and practices in waste management at working stations will help to control the person-to-person transmission of the virus. This study was conducted during the COVID-19 first wave, and we would like to recontact the participants to evaluate their behaviours at the surge of the present second wave. Alternatively, telemedicine represents the most realistic approach to provide medical assistance at sea. The same technologies respecting legal and ethical standards [37] should be considered for providing health education of seafarers.

## Figures and Tables

**Table 1:** Participant demographic and awareness characteristics.

Demographic Characteristics	N	%
Gender		
Male	1241	85.11
Female	217	14.89
Age (Years)		
<20	21	1.4
20–30	332	22.8
30–40	431	29.6
40–50	571	39.2
>60	103	7.1
Education		
Primary education	44	1.01
College/University education	1237	92.82
Secondary School education	177	6.17
Rank on board		
Deck Officers	579	39.72
Deck Rating	281	19.24
Engine Officers	268	18.35
Engine Rating	220	15.11
Galley	111	7.58

**Table 2:** Awareness on COVID-19 onboard.

Q1. Are you aware of the novel coronavirus outbreak?		
Heard from Others	13	0.90
No	18	1.23
Yes	1427	97.87
Q2. Are you or have you been infected with the novel coronavirus?		
Do not know	86	5.92
No	1365	93.63
Yes, confirmed	7	0.45
Q3. Do you know people in your immediate social environment who are or have been infected with the novel coronavirus?		
Do not know	106	7.25
No	1294	88.73
Yes, confirmed	42	2.91
Yes, not confirmed	16	1.11
Q4. Are you closely moving with other staff onboard?		
No	196	13.41
Sometimes, I cannot avoid	642	44.02
Yes	621	42.57
Q5. Was your travel history associated with infected countries in the last two months?		
Maybe	52	3.58
No	414	28.41
Yes	992	68.01
Q6. Have you used a bed previously used by someone who got infected by coronavirus?		
May be	18	1.23
No	1436	98.5
Yes	3	0.22
Q7. Which of the following is correct about the transmission of the novel coronavirus?		
Do not know	11	0.78
The novel coronavirus is not transmissible	3	0.22
The novel coronavirus is transmissible from person to person.	1435	98.44
The novel coronavirus is transmitted by animals to humans only	8	0.56
Q8. Are your handwashing for at least 20 s?		
Yes	1366	93.68
No	92	6.32
Q9. How is your mental health during these periods		
Missing family and friends	395	27.1
Feeling lonely	33	2.24
More often getting stress	168	11.53
Feeling well	862	59.13

**Table 3:** Frequency of correct answers to the knowledge questions. CKI: Cohen’s Kappa Index.

Knowledge Questions	N (Correct%)	CKI
KQ1: The main clinical symptoms of COVID-19 are fever, fatigue, shortness of breath, and dry cough.	1449 (99.4)	0.7
KQ2: Not all persons with COVID-19 will develop into severe cases. Those who are older and have chronic illnesses such as diabetes, heart diseases, cancer, and chronic kidney diseases are more likely to have severe cases.	1395 (95.7)	0.6
KQ3: The COVID-19 virus spreads via respiratory droplets of infected individuals.	1418 (97.3)	0.8
KQ4: By wearing masks onboard, it is possible to control the speed of the virus spreading.	1338 (91.8)	0.9
KQ5: Isolations and treatment of people who are infected with COVID-19 are effective ways to reduce the spread of the virus.	1443 (99)	0.8
KQ6: People who have contact with someone infected with the COVID-19 virus should be immediately isolated. In general, the observation period is 14 days.	1448 (99.3)	0.7

**Table 4:** Background characteristics of seafarers and knowledge scores of COVID-19 by age, rank, and educational status.

Demographic Characteristics				
	N (%)	Knowledge (Mean + S.D)	F-Test	*p*-Value
**Age Group**				
<20 Years	21 (1.44)	5.43 + 0.81	5.44	<0.001
20–30 Years	312 (21.4)	5.76 + 0.56		
31–40 Years	440 (30.2)	5.84 + 0.49		
41–50 Years	573 (39.3)	5.86 + 0.44		
>51 Years	112 (7.7)	5.79 + 0.60		
**Rank category**				
Deck officer	579 (39.7)	5.98 + 0.12	32.18	0.220
Engine officer	268 (18.4)	5.76 + 0.57		
Deck Rating	281 (19.3)	5.73 + 0.61		
Engine Rating	220 (15)	5.74 + 0.60		
Galley	110 (7.5)	5.51 + 0.76		
**Educational status**				
Primary school	44 (3)	5.70 + 0.55	1.52	<0.001
Secondary school	177 (12)	5.85 + 0.45		
College/University	1237 (85)	5.82 + 0.52		

## Data Availability

The COVID-19 data were extracted from the public domain data repository of the Centre for Systems Science and Engineering (CSSE) at John Hopkins University.
